# Combined neutrophil-platelet score and hemoglobin level predict survival in esophageal squamous cell carcinoma patients treated with chemoradiotherapy

**DOI:** 10.18632/oncotarget.21313

**Published:** 2017-09-27

**Authors:** Chuanwang Miao, Shan Zhu, Hong Pan, Xiaolan Cao, Shuanghu Yuan, Xudong Hu

**Affiliations:** ^1^ School of Medicine and Life Sciences, University of Jinan-Shandong Academy of Medical Sciences, Jinan, Shandong Province, P.R. China; ^2^ Department of Radiotherapy, Shandong Cancer Hospital Affiliated to Shandong University, Jinan, Shandong Province, P.R. China; ^3^ Department of Head and Neck Radiotherapy, Shandong Provincial Hospital Affiliated to Shandong University, Jinan, Shandong Province, P.R. China; ^4^ Department of Head and Neck Radiotherapy, Shandong Provincial Western Hospital, Jinan, Shandong Province, P.R. China; ^5^ Central Sterile Supply Department, Shandong Cancer Hospital Affiliated to Shandong University, Jinan, Shandong Province, P.R. China

**Keywords:** chemoradiotherapy (CRT), esophageal squamous cell carcinoma (ESCC), neutrophil-to-lymphocyte ratio (NLR), platelet-to-lymphocyte ratio (PLR), hemoglobin (Hb)

## Abstract

Systemic inflammation and hematological markers have prognostic value in patients with esophageal squamous cell carcinoma (ESCC). The objective of this study was to evaluate the neutrophil-to-lymphocyte ratio (NLR), platelet-to-lymphocyte ratio (PLR), combined neutrophil-platelet (CNP) score, and hemoglobin (Hb) to inform treatment decisions and predict outcomes in patients with locally advanced ESCC treated with chemoradiotherapy (CRT). A total of 168 patients with locally advanced ESCC were retrospectively evaluated. Patients were stratified by marker value using a receiver operating characteristic curve analysis to determine the cutoff point. Logistic regression was used to identify markers associated with sensitivity to treatment. Overall survival (OS) was calculated by the Kaplan–Meier method. Multivariate Cox logistic regression modeling was used to assess the influences of OS. Smoking history, tumour site, clinical stage, NLR, PLR, CNP, and Hb (*p* ≤ 0.05) were associated with the sensitivity to therapy. In multivariate analysis, a high CNP score was independently associated with poor treatment sensitivity (OR = 2.066, *p* = 0.021). Univariate analysis revealed that PLR, CNP, and Hb levels were associated with OS, and Cox multivariate analysis found that CNP (HR = 1.47, *p* = 0.027) and Hb level (HR = 0.44, *p* = 0.007) were independent predictors of OS. In conclusion, CNP and Hb are inexpensive and universally available prognostic markers in patients with locally advanced ESCC patients. CNP score is a systemic inflammatory marker that predicted sensitivity to CRT.

## INTRODUCTION

Esophageal cancer is prevalent in China [1], with an estimated incidence of 21.62/100,000 and estimated mortality of 16.25/100,000 population in 2011 [1]. Esophageal squamous cell carcinoma (ESCC) is the most common type of cancer, accounting for 88.8% of all diagnosed cases [1]. The incidence of ESCC has decreased over the past 20 years, but the prognosis remains very poor, with an overall 5-year survival rate of approximately 20% and only 3.8% for advanced disease [2]. Improved diagnostic and prognostic markers and individualized treatment strategies are needed to improve prognosis.

The tumor-associated inflammatory response may reflect either compromised host immune function or an antitumor immune response [3]. Systemic inflammation can be evaluated with routinely available hematological and clinical laboratory testing and is of interest as a potential marker of treatment response [3]. Studies of ESCC pathogenesis and advances in precision and personalized medicine for ESCC patients have increased efforts to identify inflammatory markers and clinicopathological characteristics with prognostic value [4]. Recent evidence has supported systemic tumor-associated inflammatory response markers, including C-reactive protein (CRP) and the Glasgow prognosis score (GPS) as able to predict the outcome in ESCC patients treated with surgery [5, 6]. Similar studies have not been conducted in ESCC patients treated with chemoradiotherapy (CRT), and CRP is not routinely evaluated in those patients.

Correlation of presurgical neutrophil-to-lymphocyte ratio (NLR) and platelet-to-lymphocyte ratio (PLR) and poor prognosis have been reported in patients with lung, liver, breast, and colorectal cancer [7–10]. Hyder et al. reported changes in the NLR and PLR during CRT that predicted survival and pathologic complete response in ESCC patients [11], but relationships of inflammatory factors and CRT sensitivity were not reported. Patients with ESCC experience physical decline because of malnutrition resulting from impaired ability to swallow and systemic effects of malignancy. A reduction in hemoglobin (Hb) in such patients has prognostic value following surgery or CRT [12–14].

Evidence of systemic inflammatory and hematological variables as markers of CRT response and outcome in patients with locally advanced ESCC is limited. We previously demonstrated that NLR, CNP, and clinical stage were significantly associated with CRT sensitivity in patients with ESCC [15]. The objective of this study was to investigate the value of NLR, PLR, CNP, and Hb in patients with locally advanced ESCC and CRT to inform treatment decisions and predict treatment outcomes.

## RESULTS

### Patient demographics

Of the 168 patients who were included, 134 were men (79.8%), 34 were women (20.2%), and their average age was 67.15 ± 8.86 years. The patient clinicopathological characteristics (gender, age, smoking history, tumor site, tumor stage) are summarized in Table 1. The clinical responses following therapy are shown in Table 2, and patient survival is reported in Table 3.

**Table 1:** Patient group and demographic characteristics

Factor	Patient (n=168)
Age(≤60/>60)	32(19.0%)/136(81.0%)
Sex (male/female)	134(79.8%)/34(20.2%)
Smoking (no/yes)	72(42.9%)/96(57.1%)
Tumour site (Upper 1/3/Middle 1/3/Lower 1/3)	60(35.7%)/78(46.4%)/30(17.9%)
T stage (II/III/IV)	24(14.3%)/106(63.1%)/38(22.6%)
N stage (0/I)	44(26.2%)/124(73.8%)
Clinic stage (II/III/IV)	44(26.2%)/86(51.2%)/38(22.6%)
Adjuvant therapies (RT/CCRT)	46(27.4%)/122(72.6%)

RT: radical radiotherapy only; CCRT: concurrent chemoradiotherapy.

**Table 2:** Univariate analyses of sensitivity to chemoradiotherapy in ESCC patients

Factor	N	Responder	Resistance	Chi-square	*p* value
Age					
≤60	32	22	10	0.523	0.505
>60	136	102	34		
Sex					
Male	134	100	34	0.229	0.665
Female	34	24	10		
Smoking					
No	72	42	30	15.611	<0.001
Yes	96	82	14		
Tumour site					
Upper 1/3	60	54	10	5.979	0.05
Middle 1/3	78	50	24		
Lower 1/3	30	20	10		
T stage					
II/III	130	98	32	0.738	0.390
IV	38	26	12		
N stage					
0	44	34	10	0.370	0.690
I	124	90	34		
Clinic stage					
II	44	34	10	17.811	<0.001
III	86	70	14		
IV	38	18	20		
Adjuvant therapies					
RT	46	30	16	2.419	0.167
CCRT	122	94	28		
NLR					
≤3.34	108	88	20	9.207	0.003
>3.34	60	36	24		
PLR					
≤103.75	68	58	10	7.795	0.005
>103.75	100	66	34		
CNP					
0 score	64	64	10	15.283	<0.001
1 score	48	38	10		
2 score	56	32	24		
Hemoglobin level					
≤43.98	80	50	30	10.105	<0.001
>43.98	88	74	14		

**Table 3:** Univariate analyses of OS in ESCC patients

	3-year OS	Log rank(Chi-square)	*p* value
Age(≤60/>60)	56.3%/44.4%	0.341	0.559
Sex(Male/Female)	46.3%/47.1%	0.012	0.912
Smocking (No/Yes)	41.7%/50.0%	0.456	0.499
Tumour site(Upper1/3, Middle 1/3, Lower 1/3)	43.3%/51.3%/40.0%	0.315	0.575
T stage (II, III/IV)	58.3%/49.1%/31.6%	2.964	0.227
N stage(0/I)	59.1%/41.9%	2.304	0.129
Clinic stage (II, III/IV)	68.2%/37.2%/42.1%	4.769	0.092
therapies (RT/CCRT)	30.4%/52.2%	0.930	0.335
NLR(≤3.34/>3.34)	60%/31.0%	2.809	0.094
PLR(≤103.75/>103.75)	58.3%/30.6%	5.491	0.019
CNP(0/1/2 score)	59.5%/47.1%/24.0%	6.078	0.048
Hemoglobin(≤43.98/>43.98)	27.6%/56.4%	8.784	0.003

### Cutoff points of potential predictive serum markers

The optimum cutoff points of the NLR, PLR, and Hb level calculated by the maximum values of the Youden index were the test variables, and treatment response was the static variable that was used to construct the ROC curve. The optimal critical values for predicting the response to therapy were an NLR of 3.34, a PLR of 103.75, and an Hb concentration of 43.98 g/L; the area under the curve (AUC) was 0.520, 0.505, and 0.505. The ROC curve was conducted for OS as a static variable. The optimal critical values associated with improved OS were an NLR of 3.51, a PLR of 145.41, and an Hb concentration of 132.50 g/L; the AUC was 0.501, 0.503, and 0.607.

### Treatment response and predictive markers

Of the 168 included patients, 124 (73.8%) had a complete response (CR) or a partial response (PR); 44 patients (26.2%) had stable disease (SD) or progressive disease (PD). The analysis of relationships between treatment response and patient variables included age, sex, smoking history, tumor location, clinical stage, NLR, PLR, CNP score, and Hb level. Univariate analysis were found significant relationships between response and smoking history (*p* < 0.001), tumour site (*p* = 0.05), clinical stage (*p* < 0.001), NLR (*p* = 0.003), PLR (*p* = 0.005), CNP (*p* < 0.001), and Hb level (*p* < 0.001) (Table 2). In subsequent logistic multifactor analysis, only a high CNP score (HR = 2.066, *p* = 0.021) was independently associated with sensitivity to therapy (Table 4).

**Table 4:** Multivariate analyses of chemoradiotherapy in ESCC patients

	*p* value	OR	OR 95%CI	
			upper limit	lower limit
CNP	0.021	2.066	1.114	3.833

### Survival outcomes and prognostic factors

**Figure 1:**
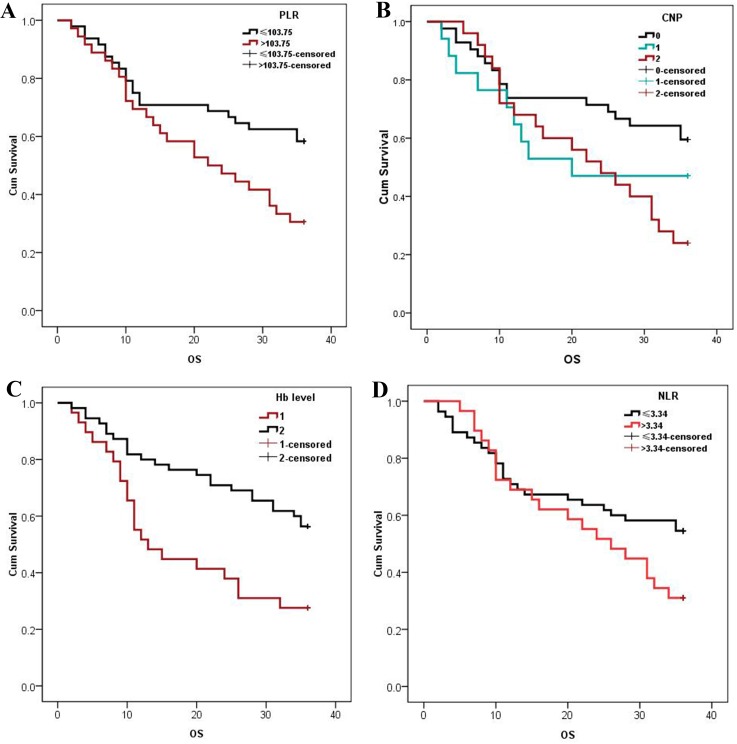
Kaplan–Meier estimates of OS of patients stratified by PLR **(A)**, CNP **(B)**, Hb level **(C)** and NLR **(D)**. Patients with PLRs >103.75, CNP scores of 3, and a Hb concentration <43.98 g/L had significantly decreased OS (P<0.05). The 3-year cumulative OS of patients with an NLR ≤3.34 was 54.5% and was 31.0% in those with an NLR >3.34.

The median OS was 33 (range 2–36) months. Three-year OS was better in patients with low (58.3%) than with high (30.6%) PLRs or low (59.5%) than with high (25.0%) CNP scores (both *p* <0.05; Figure 1A, 1B). Three-year OS was better in patients with high (56.4%) than with low (27.6%) Hb level (*p* <0.05; Figure 1C). Kaplan–Meier survival curves (Figure 1D) show that 3-year OS of patients with low NLRs (54.5%) was better than that of those with high NLRs (31.0%), but the difference was not significant.

Univariate analysis of the relationship of age, sex, smoking history, tumor location, clinical stage, NLR, PLR, CNP score, and Hb level and prognosis revealed that elevated PLR (*p* = 0.019), elevated CNP score (*p* = 0.048), and a decreased Hb level (*p* = 0.003) were associated with decreased OS. Multivariate analysis showed that elevated CNP score (HR = 1.465, *p* = 0.027), and decreased Hb level (HR = 0.444, *p* = 0.007) were both independent prognostic markers of decreased OS (Table 5).

**Table 5:** Multivariate analyses of OS in ESCC patients

	*p* value	HR	HR 95%CI	
			upper limit	upper limit
CNP	0.027	1.465	1.045	2.052
Hemoglobin	0.007	0.444	0.245	2.052

## DISCUSSION

This study is one of only a few to evaluate the prognostic value of hematological markers of systemic inflammation and nutritional state for clinical outcome for patients with locally advanced ESCC treated with CRT. Evaluation of NLR and PLR can easily be performed routinely in clinical laboratories, and univariate analysis found that reduced NLR and PLR were associated with increased effectiveness of therapy and with improved OS. The study also found that a high CNP score was an independent risk factor affecting CRT sensitivity and patient outcome. Hb level also had prognostic value. These initial findings warrant further study in larger, controlled prospective studies of ESCC patients treated with CRT to provide evidence-based guidance for the use of these laboratory tests, which are inexpensive, highly reliable, and reproducible.

The clinical outcomes of cancer patients depend on both the characteristics of the tumor itself and on host responses [16], including inflammatory and immune responses that limit the cancer development and progression [17]. Cancer cells may secrete growth factors, including granulocyte colony-stimulating factor (G-CSF) or granulocyte macrophage colony-stimulating factor (M-CSF), both of which stimulate an increase in the number of peripheral blood neutrophils [18]. Neutrophils produce tumor-associated vascular endothelial growth factor, interleukin, and tumor necrosis factor, all of which can disrupt the tumor stroma to facilitate invasion and metastasis [19]. Cancer cells may also release inflammatory mediators that stimulate megakaryocytes in the bone marrow, which results in an increase in platelets in the peripheral blood [20]. Blood platelets can also promote the invasion and metastasis of tumor cells [21]. Lymphocytes, primarily CD8^+^ T cells and NK cells, identify and clear tumor cells [22]; reduction in the number of lymphocytes weakens the antitumor immune response and increases the probability of tumor cell immune escape and becoming lost to immune system monitoring. Increased tumor growth leads to reduced CRT sensitivity and poor prognosis. Previous studies of the effects of Hb level on patient prognosis in cancer [12, 13] indicate that increased Hb levels lead to reduced tumor hypoxia and improved CRT sensitivity, thus improving the therapeutic effect [14]. The measurement of Hb levels may thus be included in a prognosis scoring system for cancer ESCC patients undergoing CRT [23].

Previous studies that evaluated the association of NLR, PLR, and patient outcome in 1,540 patients with esophagectomy [24–29] reported that elevated NLR and PLR were associated with poor prognosis. However, less data are available in patients not indicated for surgery and treated by CRT. Multivariate analysis in this patient series showed that PLR and NLR alone were not independent predictors of patient outcome. Previous studies of the GPS, which includes the assay of CRP, found that it was associated with reduced survival of ESCC patients [6]. However, CRP is not routinely included in routine laboratory testing prior to CRT. In this study, CNP score was an independent predictor of patient outcome and CRT sensitivity. In the evaluation, CRT sensitivity and variables with a significant effect in univariate analysis were included in a multivariate logistic regression analysis, which confirmed that a high CNP score was independently associated with sensitivity to therapy (OR = 2.066, *p* = 0.021), and Cox multifactorial model analysis found that CNP score was an independently associated with improved OS (OR = 1.47, *p* = 0.027).

The measurement of Hb levels is routinely performed for all patients on admission to the hospital. Although it has low specificity, reduced Hb levels are often found in patients with cancer, and may indirectly reflect nutritional status. In a series of 103 ESCC patients with radiotherapy and concurrent chemotherapy, Zhang et al. found that 3-year OS was reduced in patients with anemia [23]. In this study, patients with normal or high Hb levels had an improved sensitivity to CRT, and the Cox multifactorial model found that Hb was an independent predictor of OS. Therefore, early individualized treatment and nutritional support may have both short-term efficacy and long-term survival benefit.

The study limitations include its single-center retrospective design and small patient sample size. Also, the predictive value of the inflammation-associated serological factors such as the GPS could not be evaluated because they are not performed in routine clinical examinations. Histological evaluation of the relationship between tissue and systemic neutrophilia to confirm that neutrophilia was a consequence of the tumor, rather than another factor, such as infection, could not be done. Larger, controlled prospective clinical studies are warranted to confirm the predictive value of these inflammatory markers on the response to CRT and on the long-term survival of patients with locally advanced ESCC. In conclusion, despite the acknowledged limitations, CNP score offers additional sensitivity to the choice of CRT for the clinical management of locally advanced ESCC. CNP score and Hb level had prognostic value in locally advanced ESCC. This analysis showed that a low CNP score was the risk factor and high Hb level appeared to be protective.

## MATERIALS AND METHODS

### Patients

This retrospective study included patients with locally advanced ESCC with CRT at Shandong Cancer Hospital Affiliated to Shandong University between 2006 and 2012. Patients with histologically confirmed ESCC treated with CRT, available laboratory and hematological testing results within 7 days of treatment, and available clinicopathological and follow-up data were eligible. ESCC patients with acute or chronic infectious disease or disease-associated complications; targeted antitumor therapy; liver, kidney, or autoimmune disease; thrombosis or bleeding disorders were excluded. All patients reviewed the study protocol and gave written informed consent before participation. The Ethics Committee of Shandong Cancer Hospital Affiliated to Shandong University approved the study.

### Evaluation of inflammation markers

Pretreatment white blood cell, neutrophil, lymphocyte, monocyte, platelet counts, and Hb level were included in the data analysis. The NLR was calculated by dividing the absolute neutrophil count by the absolute lymphocyte count. The PLR was calculated by dividing the absolute platelet count by the absolute lymphocyte count. Patients with reduced NLRs and PLRs were given a combined neutrophil-platelet (CNP) score of 0. Those with a reduced NLR or PLR were given a CNP of 1, and those with normal NLRs and PLRs were given a score of 2.

### Treatment

The radiotherapy protocol included a total radiation dose of up to 59.6 Gy delivered by standard fractionated radiotherapy in 34 fractions on weekdays; 2.0 Gy/f × 20f, 1.4Gy/f × 14f; over a 6-week cycle. Concurrent chemotherapy consisted of 2 28-day cycles of daily cisplatin (75–100 mg/m^2^/dL) and fluorouracil (750–1000 mg/m^2^/dL).

### Clinical evaluation and follow-up

Treatment evaluation followed the Response Evaluation Criteria In Solid Tumors criteria [30]. Patients with a complete response (CR) or partial response (PR) following treatment were “responders.” Patients exhibiting stable disease (SD) or progressive disease (PD) were classified as “resistant”. Follow-up evaluations were performed every 3 months after patients were discharged from the hospital and included a medical history, physical examination, and computed tomography of the chest. Endoscopy was performed in cases of clinical evidence of recurrence or metastasis. The last follow-up visits were in February 2015. Survival period was measured from the date of admission to the date of death, or to the date of the last follow-up.

### Statistical analysis

The study data analyzed as possible predictive markers were sex, age, smoking history, tumor site, tumor stage, NLR, PLR, CNP, and Hb. Optimal cutoff values were determined by the maximum potential effectiveness values of the Youden index, a summary measure of the receiver operating characteristic (ROC) curve [31]. The chi-square test was used to analyze the relationship between possible predictive markers and clinical responses following therapy. Variables with *p*-values ≤0.05 by univariate analysis were included in multivariate logistic regression analysis. Overall survival (OS) was calculated by the Kaplan–Meier method, and the significance of differences was determined by the log-rank test. Multivariate Cox logistic regression modeling was used to assess the influence of predictive markers on patient survival outcomes. Statistical significance was defined as *p* ≤ 0.05 or a 95% confidence interval that did not exceed 1.00. All analyses were conducted using SPSS 17.0 software.
